# Highly Tough, Stretchable and Self-Healing Polyampholyte Elastomers with Dual Adhesiveness

**DOI:** 10.3390/ijms23094548

**Published:** 2022-04-20

**Authors:** Pengfei Yin, Yang Liu, Dan Huang, Chao Zhang

**Affiliations:** Department of Biomedical Engineering, Sun Yat-Sen University Shenzhen Campus, Shenzhen 518107, China; yinpf3@mail2.sysu.edu.cn (P.Y.); huangd48@mail2.sysu.edu.cn (D.H.)

**Keywords:** polyampholytes, tough, stretchable, self-recovery, self-healing, dual adhesiveness

## Abstract

A new type of polyampholyte with unique viscoelastic properties can be easily synthesized by the copolymerization of butyl acrylate with dimethylaminoethyl methacrylate and acid acrylate in one pot. The elastic modulus of the as-prepared polyampholyte can be easily tuned by adjusting the ratio between the butyl acrylate and ionic monomers. The polyampholyte synthesized by a low proportion of ionic monomer showed low tensile strength and high stretchability, resulting in good conformal compliance with the biological tissues and potent energy dissipation. Due to the existence of high-intensity reversible ionic bonds in the polymer matrix, excellent self-recovery and self-healing properties were achieved on the as-prepared polyampholytes. By combining the high Coulombic interaction and interfacial energy dissipation, tough adhesiveness was obtained for the polyampholyte on various substrates. This new type of polyampholyte may have important applications in adhesives, packaging and tissue engineering.

## 1. Introduction

Polyampholytes, in which both positive and negative charges exist, have aroused widespread interest due to their tunable properties and functions, e.g., anti-fouling [1], self-healing [2,3,4] and shape memory [5]. Much attention has been given to the fabrication of polyampholytes, e.g., free radical solution polymerization [6], reverse phase micro-emulsion polymerization [7], active anionic polymerization [8] and group transfer polymerization [9]. Polyampholyte hydrogels can be formed when polyampholytes with a suitable degree of crosslinking are swollen in water, and they may find a wide range of applications in the field of biomedical engineering [10,11], tribology [12,13] and anti-fouling [14]. Due to their unique zwitterionic molecular structures and tunable interchain ionic interactions, polyampholyte hydrogels have shown outstanding self-healing, adaptive adhesiveness [15,16] and shape-memory properties. These properties are very important for the applications of polyampholyte-based hydrogels in flexible electronics, artificial skins and tissue engineering. Compared with double-network hydrogels [17,18,19], polyampholyte hydrogels may have intrinsic advantages in forming strong adhesion to different charged surfaces due to their unique self-adjustable surface charge distribution. However, polyampholytes synthesized by the direct copolymerization of anionic and cationic monomers are physically crosslinked by ionic bonds, resulting in physical gels with limited elastic moduli and toughness [20]. This in turn lowers the upper limit of the energy dissipated during the detachment between the polyampholyte and the substrate, and hence attenuated adhesion toughness. To address this issue and obtain polyampholytes with high adhesion toughness, recent attempts have been focused on introducing new monomers with zero charge into the polymeric structure of polyampholytes, but the true effect was ambiguous and essentially depended on the reactivity between the neutral and ionic monomers [21].

Herein, we proposed a new type of highly tough and adhesive polyampholyte (PBADMA) by using butyl acrylate (BA) to copolymerize the ionic monomers of dimethylaminoethyl methacrylate (DMA^+^) and acid acrylate (AA^−^) in a simple and fast one-pot manner. The as-obtained polyampholyte showed typical viscoelastic properties with high tensile strength (4.7 MPa) and an extremely high fracture strain of 2700%. The presence of BA can effectively tune the viscoelastic behaviors of the as-prepared polyampholytes, achieving excellent self-recovery and self-healing properties. High adhesion toughness surpassed the conventional hydrogel materials by means of combining the Coulombic surface interaction and viscoelastic energy dissipation was also achieved for the as-prepared polyampholytes.

## 2. Results

### 2.1. Characterization of PBADMA

**Figure 1:**
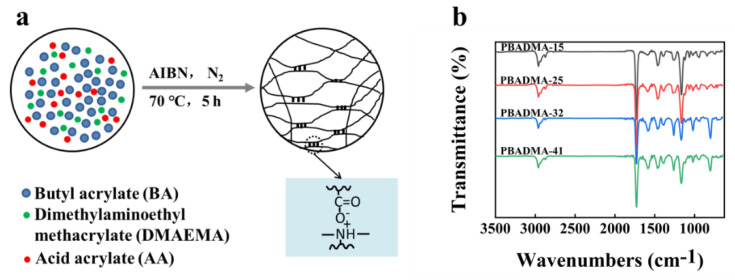
(**a**) Schematic illustration of the formation process of PA and (**b**) FT-IR spectra of the as-prepared PA elastomers.

A series of PA elastomers was prepared using BA, AA, and DMA, denoted as P(BA-co-AA-co-DMA)-x (PBADMA-x), while x represented the molar ratio of the ionic monomers. We used BA because it can be easily copolymerized with the other molecular components upon heating, and its long alkyl side chain may endow the copolymers with high chain mobility, which is beneficial for the self-healing and adhesive properties. AA and DMA were used to form a physical crosslinking structure by the electrostatic interactions of the opposite charges to strengthen the PA elastomers (Figure 1a). The chemical structure of the PA elastomer was confirmed by FT-IR date and ^1^H NMR analysis (Figure S1). The PA elastomers showed similar FT-IR information in Figure 1b and the peak near 1580 cm^−1^ was assigned for the COO^−^ antisymmetric stretching vibration of the ionized -COOH as a result of the ionic interaction between AA and DMA [22]. The intensity of the peak at 1580 cm^−1^ was found to be proportional to the molar fraction of the ionic monomers. These results confirmed that ionic interactions were formed between AA and DMA and become stronger as the molar fraction of ionic monomers is increased.

### 2.2. Mechanical Properties of PBADMA

**Figure 2:**
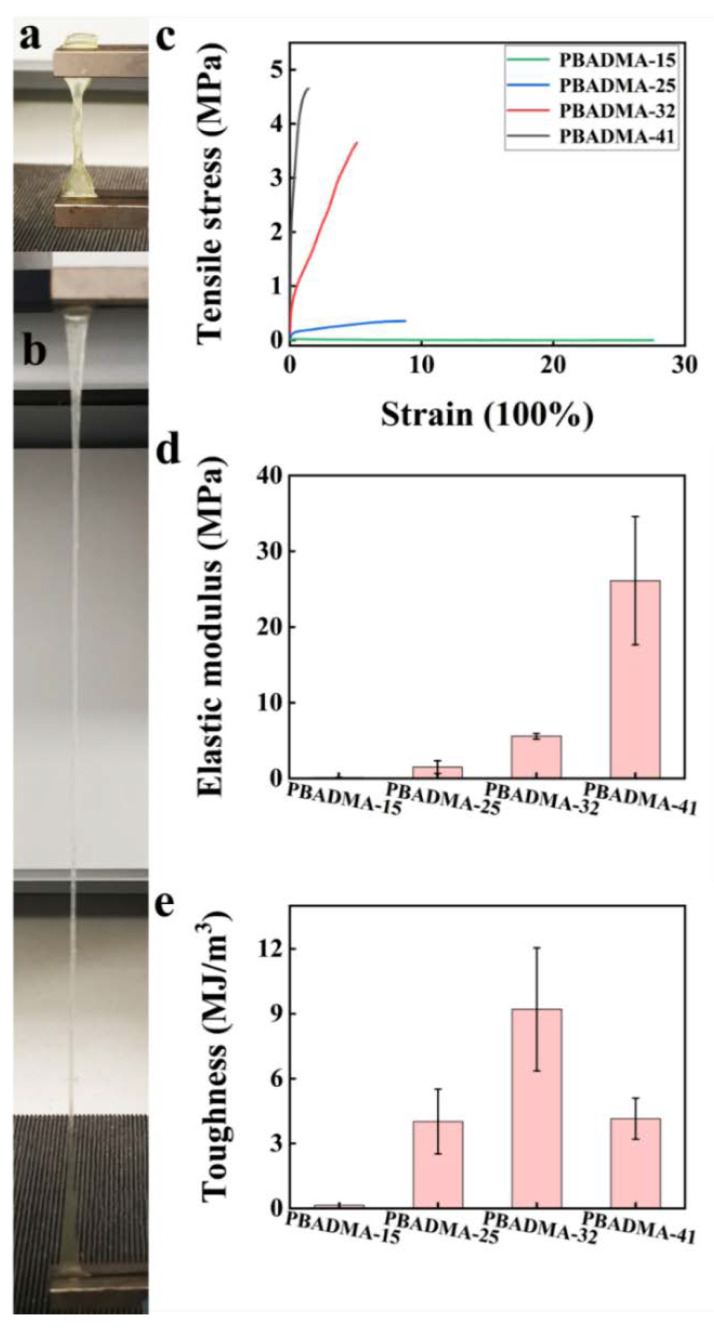
PA elastomers show extraordinary mechanical and tough properties. The optical photograph of PBADMA-15 before (**a**) and after (**b**) extended. The stress–strain curve (**c**), elastic modulus (**d**) and toughness (**e**) of PA elastomers.

The mechanical properties of PA elastomers are shown in Figure 2. It is shown that the tensile strength of PBADMA-15, PBADMA-25, PBADMA-32 and PBADMA-41 is 74 KPa, 350 KPa, 3.6 MPa and 4.7 MPa, respectively. However, the fracture strain of PBADMA-15, PBADMA-25, PBADMA-32 and PBADMA-41 is 2761%, 877%, 510% and 142%, respectively. The mechanical properties of PA elastomers are essentially dependent on the molar percentage of oppositely charged monomers in the precursor. As the molar percentage increased from 15 to 41%, the tensile strengths of the as-obtained PA were considerably increased from 0.07 to 4.7 MPa, and the corresponding ruptured strains were changed from 2761% to 142%, respectively. As the molar percentage of AA and DMA in the precursor was higher than 41% (Figure 2a–c), the PA elastomers could not be formed due to the strong electrostatic interaction between AA and DMA, resulting in the formation of milky precipitates (Figure S2). Figure 2d,e show the elastic modulus and toughness of the PA elastomers calculated from the corresponding stress–strain curves. It can be seen from Figure 2d that the elastic modulus of PA is proportional to the molar percentage of the ionic monomers. As the molar percentage of ionic monomers increased from 15% to 41%, the elastic modulus of PA significantly increased from 0.11 ± 0.03 MPa to 26.09 ± 8.47 MPa, indicating the intensification of strong ionic bonds in the PA physical gel. On the other hand, the toughness of the PA elastomers was calculated to be 0.14 ± 0.06 MJ m^−^^3^, 4.02 ± 1.51 MJ m^−^^3^, 9.2 ± 2.85 MJ m^−^^3^ and 4.15 ± 0.95 MJ m^−^^3^, respectively (Figure 2e). However, the discrepancy observed in the elastic modulus and toughness trends indicated that there exists a trade-off between the tensile strength and fracture toughness of PA upon adjusting the molar concentration of the ionic monomers. It has been reported that the strong ionic bonds may constitute the stiff network of PA, which provides high tensile strength and shape-memory property; the weak ionic bonds may constitute the tough network of PA, which endows PA with a high strain and recovery property [23]. These two networks combined form the biphasic continuous microstructure of PA, which appeared as a transparent, homogeneous elastomer. To achieve the optimal self-healing property, it is vital to manipulate the relative proportion of the strong and weak bonds. It is believed that by conducting copolymerization in the atmospheric condition, the participation of free oxygens in the reaction can reduce the crosslinking density of the polyampholytes, forming products with low elastic modulus.

**Figure 3:**
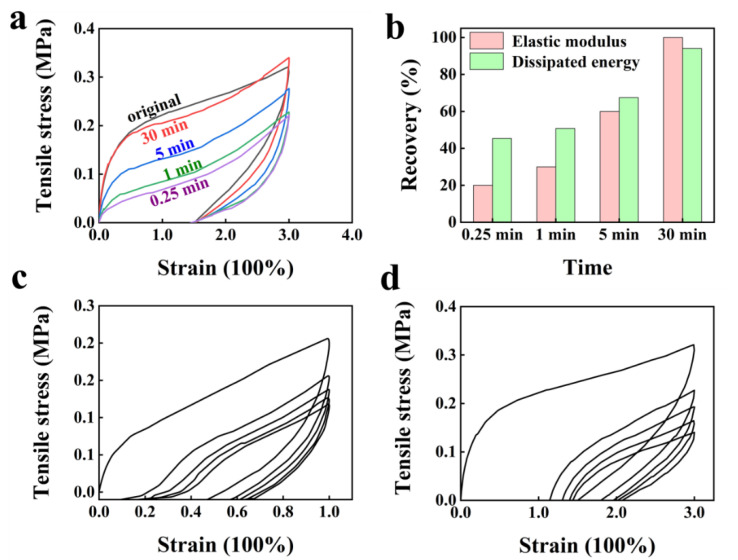
Loading–unloading test under various conditions: (**a**) loading–unloading tests of PBADMA-25 under fixed strain of 300% after different rest times (0.25 min, 1 min, 5 min and 30 min); (**b**) the elastic modulus and dissipated energy calculated from (**c**). Five-cycle loading–unloading tests of PBADMA-25 under different strains of 100% (**c**) and 300% (**d**).

By adjusting the molar percentage of the ionic monomers to 25%, PA with good self-recovery and anti-fatigue properties can be obtained, as shown in Figure 3. The as-prepared PBADMA-25 subjected to 300% strain showed a nearly complete recovery of elastic modulus and dissipated energy after resting for 30 min (Figure 3a,b). The as-prepared PBADMA-25 showed typical viscoelastic behaviors by cycling under constant strain (100% and 300%). The large hysteresis of the strain was observed for the samples in the second cycle, but the hysteresis for each subsequent cycle became smaller as the cycling test proceeded, as shown in Figure 3c; as the applied strain increased to 300%, the hysteresis observed for each subsequent cycle increased, compared to the case of the 100% strain (Figure 3d). This thus indicated that the energy dissipation during the strain for PBADMA-25 largely occurred through the breaking of the sacrificial weak ionic bonds [24]. At high strain rates, the breaking speed of the weak ionic bonds overwhelms the recovery speed of weak ionic bonds, resulting in enhanced hysteresis. The hysteresis behavior of PBADMA-25 is in good accordance with the viscoelastic model proposed by Creton et al. [25].

### 2.3. Self-Healing Property of PBADMA

**Figure 4:**
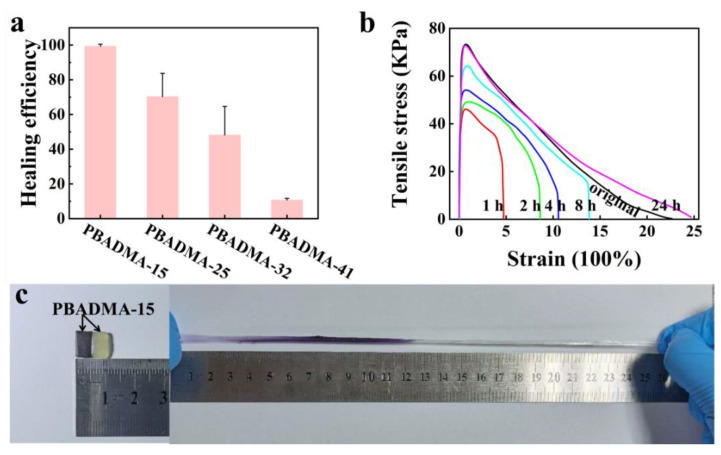
Self-healing properties of PBADMA-15: (**a**) the stress–strain curves of the samples after a different self-healing time (1 h, 2 h, 4 h, 8 h and 24 h); (**b**) healing efficiency of the PA elastomers; and (**c**) optical images of the healed sample before and after stretching.

Due to the existence of high-proportion dynamic weak ionic bonds in the gel matrix, the as-prepared PBADMA showed good self-recovery and anti-fatigue properties under high cycling strain. On the other hand, they may also show excellent self-healing properties after rapture. The self-healing properties of the PBADMA samples were shown in Figure 4a. It can be observed that the healing efficiency of the PBADMA samples decreased as the molar percentage of the ionic monomers was increased. The PBADMA sample with the lowest ionic monomer content (PBADMA-15) showed the best self-healing efficiency as a result of the high proportion of weak ionic bonds in its gel chemical structure. Figure 4b shows typical stress–strain curves of raptured PBADMA-15 samples processed for a different self-healing time. After 24 hours’ self-healing at room temperature, the tensile strength of the healed PBADMA-15 could be fully recovered, and its fracture strain was enhanced compared to the pristine state. A photograph of the self-healed PBADMA sample was shown in Figure 4c. One of the raptured parts of the sample was dyed purple to better demonstrate the effect of self-healing. Actually, the healed sample can be stretched to a strain even higher than the pristine sample. It thus evidenced that PBADMA-15 had a remarkable self-healing property.

### 2.4. Adhesion Properties of the PA Elastomers

**Figure 5:**
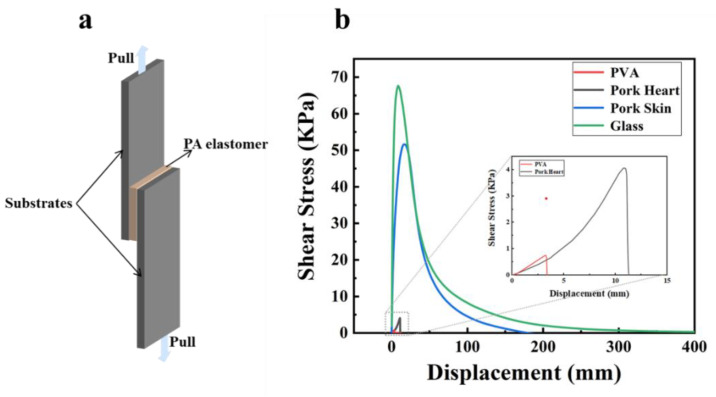
Lap shear test to measure the adhesion of PBADMA-15 to different substrates: (**a**) schematic illustration of the test; and (**b**) shear stress–displacement curves of PBADMA-15 with respect to the measured substrates.

PBADMA-15 also showed a high adhesion property to different substrates, i.e., inorganic, organic and biological (Figure S3). The lap shear measurements were used to evaluate the adhesion strength of PBADMA-15 to different substrates, including glass slide, PVA hydrogel, pork skin and pork heart; the schematic illustration of the experiment is shown in Figure 5a. In Figure 5b, the adhesion strengths of PBADMA-15 to the glass slide and pork skin are shown to be 68 and 52 KPa (Figure 5b), respectively, which are significantly higher values than those previously reported for adhesive hydrogels [26,27,28,29], as well as higher than that of commercially available glue (15 KPa). On the other hand, PBADMA-15 showed a lower adhesion strength to the substrates in the wet state, including the pork heart (4 KPa) and PVA hydrogels (0.7 KPa). In contrast to the conventional polyampholytes and elastomers, which generate adhesive force through Coulombic interactions and interfacial energy dissipation [30,31], the as-prepared viscoelastic polyampholytes, i.e., PBADMA-15, obtained the advantages of both polyampholytes and elastomers in the case of adhesion. By adjusting the molar percentage of the ionic monomers to 15%, the self-ionic association within the polyampholyte matrix can be effectively mitigated, forming a viscoelastic neutral polyampholyte with a mechanical strength of (0.07 MPa) in the same order of magnitude with the pork heart tissue (~0.024 MPa). Due to the equal molar ratio between the positively charged and negatively charged monomers, the total charge of the as-prepared PBADMA-15 is balanced to neutral, making it possible to form Coulomb interactions with all types of charged surfaces through a self-adaptive charge redistribution process [30]. Moreover, the viscoelastic PBADMA-15 can dissipate a great amount of energy during peeling, and significantly enhances the adhesion toughness. These synergistic effects make PBADMA-15 show superior adhesion strength compared to double-network hydrogels, plastic-like hydrogels and elastomers [32,33,34].

**Figure 6:**
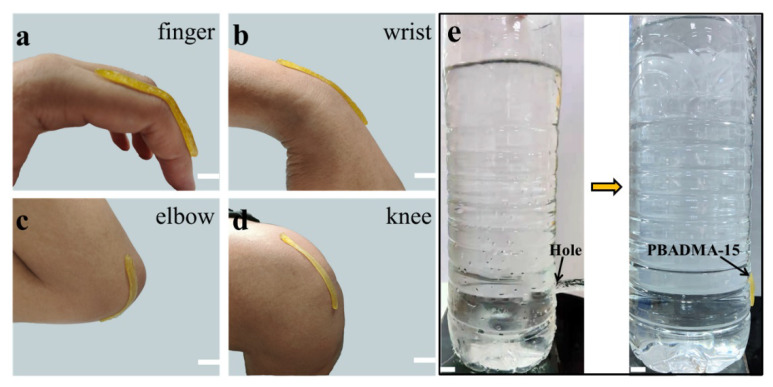
Evaluation of the dynamic adhesion properties of the as-prepared PA: (**a**–**d**) PBADMA-15 was conformally adhered to the skins of body joints during motion; (**e**) PBADMA-15 was applied onto the opened hole of a plastic bottle (PET) to prevent water leakage. Scale bar: 10 mm.

To evaluate the dynamic conformal adhesiveness of the as-prepared PA during motion, 2 mm-thick PBADMA-15 stripes were applied onto the skins of body joints, i.e., the finger knuckle, wrist, elbow, and knee, as shown in Figure 6a–d. It was observed that the PA stripe can firmly adhere to the skin surface during repeatedly joint motion, and the motion was not restricted by the stripe due to its low modulus and high stretchability. The tough adhesion property of PBADMA-15 was further demonstrated by fixing a plastic bottle with an opened hole (Figure 6e). A plastic bottle with an opened hole at the bottom was used to contain water: as can be observed from Figure 6e, water can rapidly escape from the bottle through the opened hole, but after fixing the hole by adhering a piece of PBADMA-15 onto it, no further leakage can be observed—even when the bottle was filled with water (h = 150 mm), indicating that PBADMA-15 can withstand at least 1.47 kPa water pressure without detachment.

## 3. Materials and Methods

### 3.1. Materials

Butyl acrylate (Alfa Aesar, Shanghai, China, purity > 98%), dimethylaminoethyl methacrylate (Macklin, Shanghai, China, purity 99%), acid acrylate (Damao Chemical Reagent Factory, Tianjin, China, analytical reagent), ethyl acetate (Macklin, Shanghai, China, purity 99%), methyl benzoate (Alfa Aesar, Shanghai, China, purity 99%), azobisisobutyronitrile (Macklin, Shanghai, China, purity 98%), polyvinyl alcohol (Macklin, Shanghai, China, PVA-1799), and petroleum ether (Guangzhou Chemical Reagent Factory, Guangzhou, China, analytical reagent).

### 3.2. Synthesis of PBADMA

The polyampholytes were prepared by one-pot polymerization method. Firstly, the positive charged monomer and the negative charged monomer (the molar ratio of the monomers was 1:1) were dissolved in 50 mL ethyl acetate, followed by adding butyl acrylate and methyl benzoate into the mixed solution. Afterwards, the initiator (1‰ of the total mole of monomers) was dissolved in the mixed solution. Eventually, the reaction was allowed to proceed in the mixed solution at 70 °C for 5 h under constant magnetic stirring. As the reaction was completed, the mixed solution was thoroughly centrifuged by petroleum ether at least three times. The precipitation was dried at 40 °C overnight to collect the polyampholytes. By adjusting the molar ratio of the positively and negatively charged monomers to BA, polyampholytes with different mechanical properties can be obtained.

### 3.3. Characterizations of PBADMA

#### 3.3.1. Fourier Transform Infrared Spectroscopy (FT-IR)

The FT-IR spectra of polyampholytes with different compositions were measured at room temperature by NICOLET 6700 (Thermo Scientific, Waltham, MA, USA) in the attenuated total reflection (ATR) mode to study the formation process of the polyampholytes. Pristine polyampholytes in the solid state were used for the measurement. The scan range was set between 400 cm^−1^ and 4000 cm^−1^, the resolution was set as 2 cm^−1^, and the scan number was set as 32.

#### 3.3.2. H NMR Spectroscopy

After 10 mg PA elastomer was completely dissolved in 500 μL deuteroxide in a ^1^H NMR cube, the ^1^H NMR spectra of the as-prepared samples were obtained by nuclear magnetic resonance spectrometer (Avance III, Bruker Corporation, Rheinstetten, Germany). Data analysis was carried out using the Mnova (version 14.2.2, Santiago de Compostela, Spain) to calculate the molar ratio of both positively and negatively charged monomers in the polyampholytes.

#### 3.3.3. Characterization of the Mechanical Properties

Mechanical tests of polyampholytes were performed on a LR5K Plus machine (LLOYD, West Sussex, UK) with a 100 N load cell at 100 mm min^−1^ cross head speed at room temperature. The polyampholyte samples were cut into dumbbell shape with a gauge length of 75 mm, a width of 4 mm and a thickness of 1 mm for the uniaxial tensile tests. At least five specimens were tested for each type of sample. The stress–strain curves were obtained and the fracture strength and fracture elongation of the samples were calculated. Young’s modulus (E) was obtained from the average slope of the linear region of the stress–strain curve. The fracture energy (W) or toughness was calculated from the area of the stress–strain curves by the following equation [35]:

W = ∫τ∂ε,(1)

where τ is the stress and ∂ε is the partial derivative of the strain.

Cyclic and consecutive mechanical tests were performed on the polyampholyte samples to study their energy dissipation behaviors. Cyclic tests were performed with constant strain and the consecutive tests were performed using gradient-increased strains. Strains of 100%, 200% and 300% were used, respectively. The dissipation energy was calculated from the area of the corresponding loading–unloading curve after different rest times. The recovery ratio was defined as the ratio between the hysteresis energy of the specific cycle and the first cycle.

#### 3.3.4. Characterization of the Self-Healing Property

In the self-healing experiment, the PA samples were cut into two pieces at the mid-point. Then, the two pieces of the sample were pinched together for different durations (1 h, 2 h, 4 h, 8 h and 24 h) at room temperature. Afterwards, the tensile strengths of the self-healed samples were measured. The self-healing efficiency (λ) of polyampholyte was calculated by the following equation:

λ = W_t_/W_0_, (2)

where W_t_ and W_0_ are the fracture energy of the original and self-healed samples, respectively. The self-healing efficiency for each type of PA was obtained by three parallel experiments.

#### 3.3.5. Characterization of the Adhesion Properties

The adhesive property of the PA elastomers was measured by the lap shear test, a process that was generally used for measuring the adhesive strengths of the hydrogel-based tissue adhesives [36,37,38]. Two substrates (length × width = 75 mm × 25 mm) were brought into contact with a piece of PA elastomer (length × width × height = 20 mm × 25 mm × 1.7 mm), forming a junction contact area of 5 cm^2^. The lap joint was slightly pressurized with a finger for 30 s, then the two ends of the glass substrates were clamped to the tensile machine. The shear adhesive test was performed at a shear velocity of 100 mm min^−1^. The substrates used in this study were glass slides, PVA hydrogel, pork skin and pork heart, respectively.

## 4. Conclusions

In conclusion, a new type of polyampholytes with unique viscoelastic properties can be easily synthesized in a one-pot manner by copolymerizing butyl acrylate with ionic DMA and AA. The tensile strength and fracture strain of the as-prepared polyampholytes can be easily tuned by changing the proportion between the butyl acrylate and ionic monomers for a tensile strength as high as 4.7 MPa and a fracture strain as high as 2761% can be achieved, respectively. By lowering the molar proportion of the ionic monomers, polyampholytes of low tensile strength and high fracture strain can be obtained, which showed remarkable self-recovery and self-healing properties. This phenomenon can be attributed to the high density of weak ionic bonds in the polymeric matrix of the as-prepared polyampholytes, which may act as reversible sacrificial bonds to dissipate energy during stretching. The viscoelastic polyampholytes can realize strong and reversible adhesion to a wide range of substrates with different surface charges, owing to both the surface charge self-adjustability of polyampholytes and the intensive energy dissipation originating from the viscoelasticity. The as-prepared polyampholyte showed tough adhesions as body stripes and water stoppers, indicating its potent applications in adhesives, packaging and tissue engineering.

## Data Availability

The data presented in this study are available on request from the corresponding author.

## Supplementary Materials

The supporting information can be downloaded at: https://www.mdpi.com/article/10.3390/ijms23094548/s1.
