# Supplementary material for: Highly Tough, Stretchable and Self-Healing Polyampholyte Elastomers with Dual Adhesiveness
Source: Int J Mol Sci. 2022 Apr 20;23(9):4548. doi: 10.3390/ijms23094548 (PMC9104851; doi:10.3390/ijms23094548)
Supplement: Supplementary file 1 [file ijms-23-04548-s001.zip › ijms-1687794-supplementary.pdf]

Supporting Information

Highly tough, stretchable and self-healing polyampholyte elastomers with dual adhesiveness

Pengfei Yin, Yang Liu, Dan Huang and Chao Zhang**

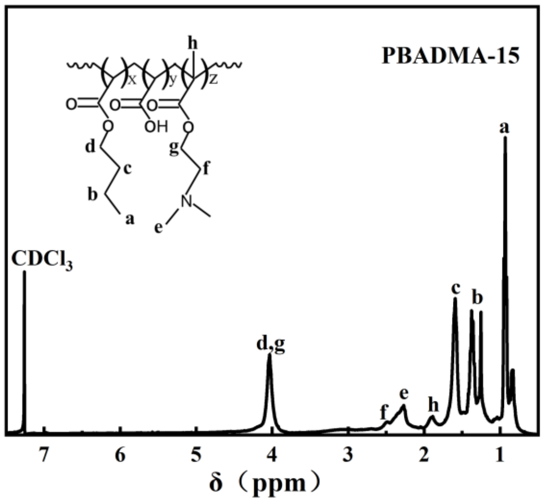

Figure S1. The 1H NMR spectrum of PBADMA-15.

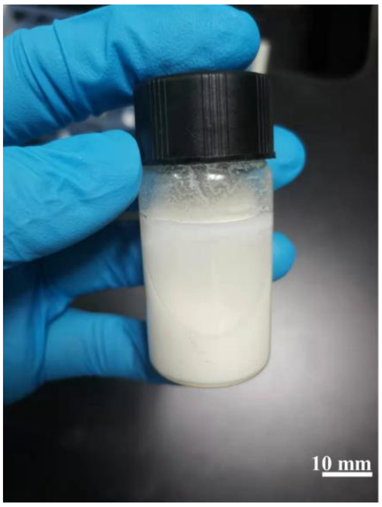

Figure S2. Milky precipitates were observed when the molar percentage of AA and DMA in the precursor was higher than 41%.

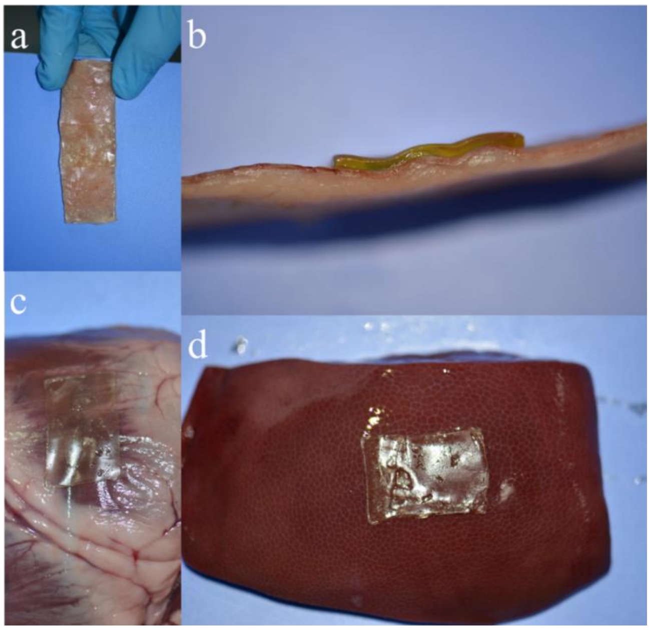

Figure S3. PBADMA-15 shows good adhesion to pork tissues, including: (**a**) Pork skin; (**b**) shows the lateral view of (**a**); (**c**) Pork heart and (**d**) Pork liver.
